# Supplementary figures and images for: TSLPR deficiency attenuates AHR independently of eosinophilia and mucus secretion in a chronic HDM mouse model of allergic asthma
Source: PLoS One. 2025 Nov 19;20(11):e0335742. doi: 10.1371/journal.pone.0335742 (PMC12629416; doi:10.1371/journal.pone.0335742)

**A**

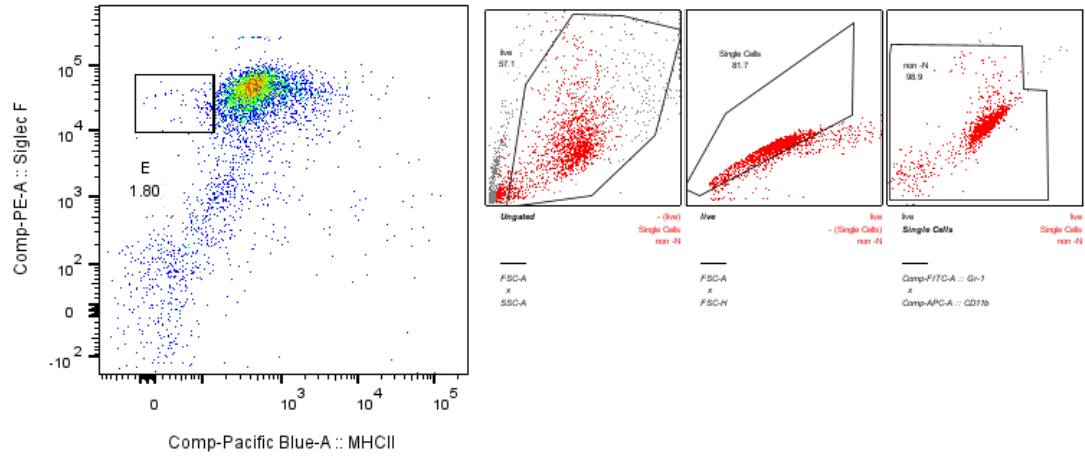

**B**

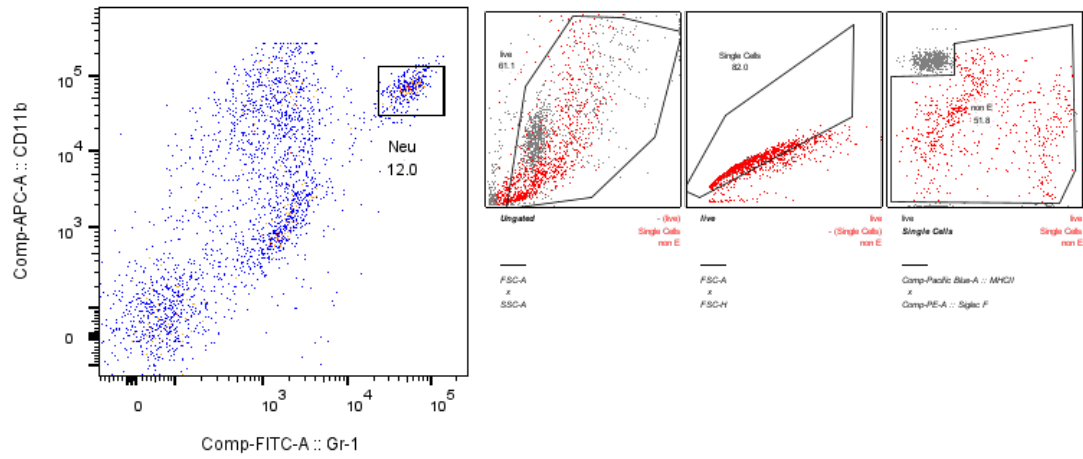

Supplement: S1 Fig — (A) Eosinophils were identified by gating on Siglec-F⁺ and MHCII⁻ cells. Initial gating steps included selecting live, single cells to exclude debris and doublets, followed by gating on Siglec-F and MHCII expression to isolate the eosinophil population. The representative sample shown is from the HDM-exposed group, selected due to the higher number of cells recovered in BALF, allowing for clearer visualization of the gating strategy. (B) Neutrophils were identified by gating on CD11b⁺ and GR1 ⁺ cells, with eosinophils excluded. (PDF) [file pone.0335742.s001.pdf]

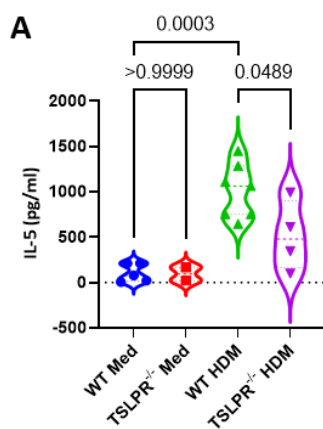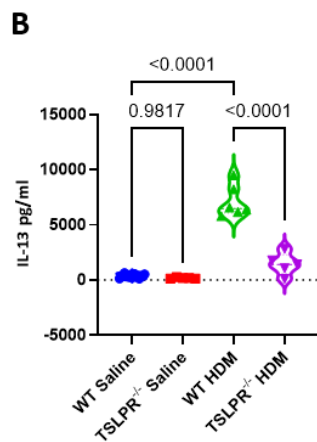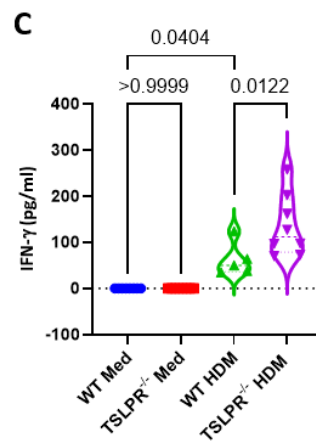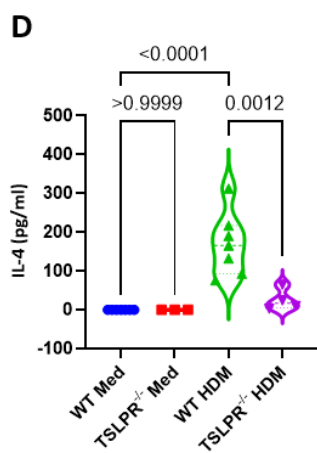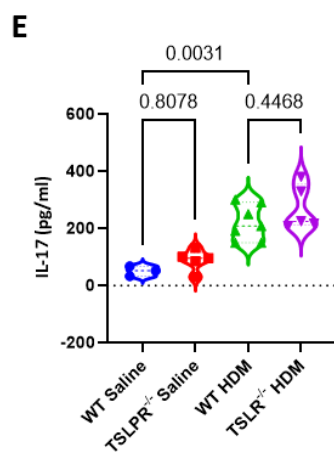

Supplement: S2 Fig — Single-cell suspension of mediastinal lymph node from the mice exposed to HDM or saline was prepared and stimulated with medium or HDM in vitro. The levels of IL-4, IL-5, IL-13, IL-17A, and IFN-γ were measured in the supernatant of the cultured cells after 72 hours by ELISA (n = 3–8 per group). *p < 0.05, **p < 0.01, and ***p < 0.001 (one-way ANOVA). Violin plots display the data distribution with median and interquartile range. Results are from three independent experiments. (PDF) [file pone.0335742.s002.pdf]

**A**

**WT** **Saline** ***TSLPR*<sup>-/-</sup>**

1 2 3 4 5 6 7 8 9

**Collagen 1**

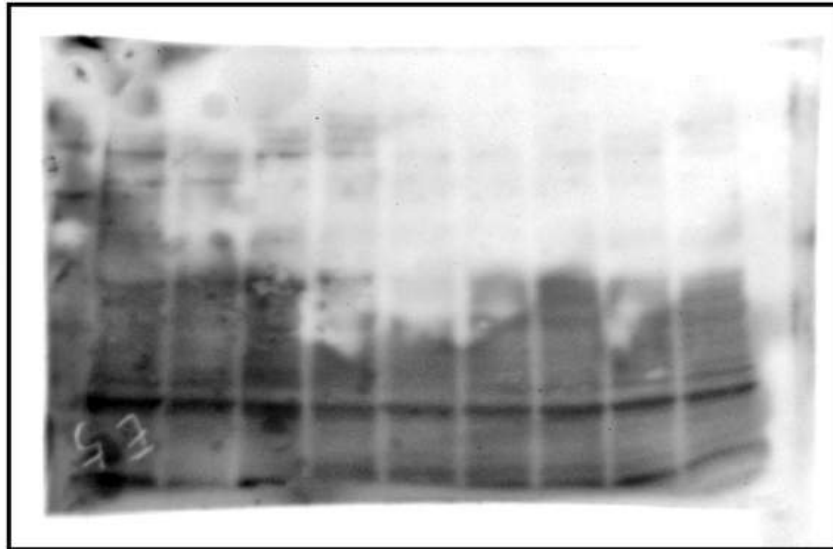

**GAPDH**

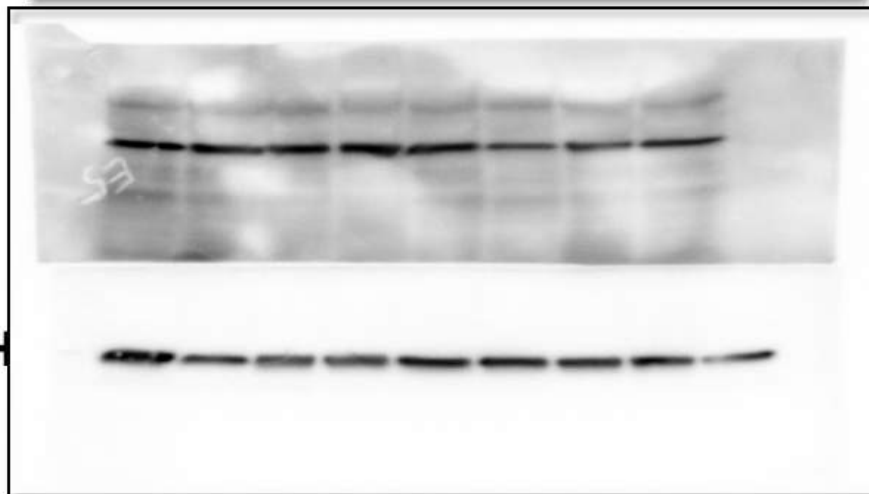

**WT** **HDM** ***TSLPR*<sup>-/-</sup>**

1 2 3 4 1 2 3 4

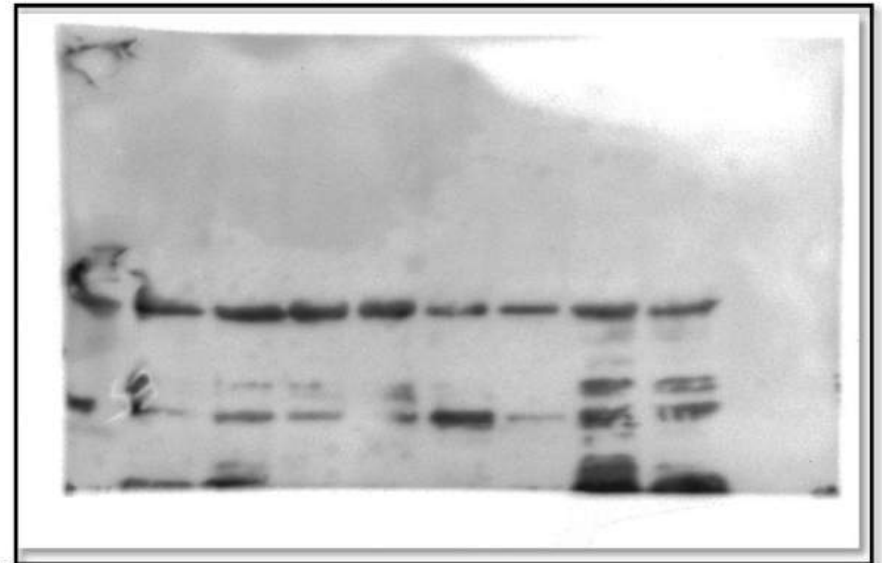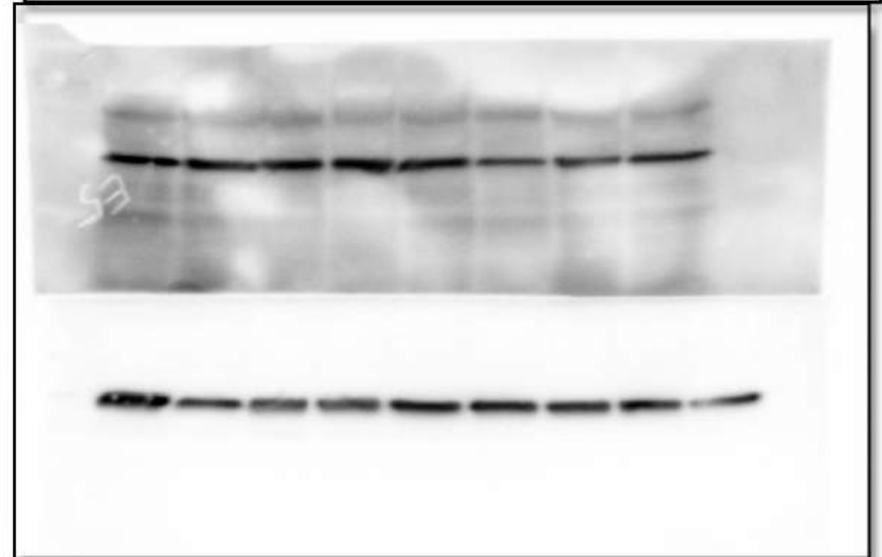

**B**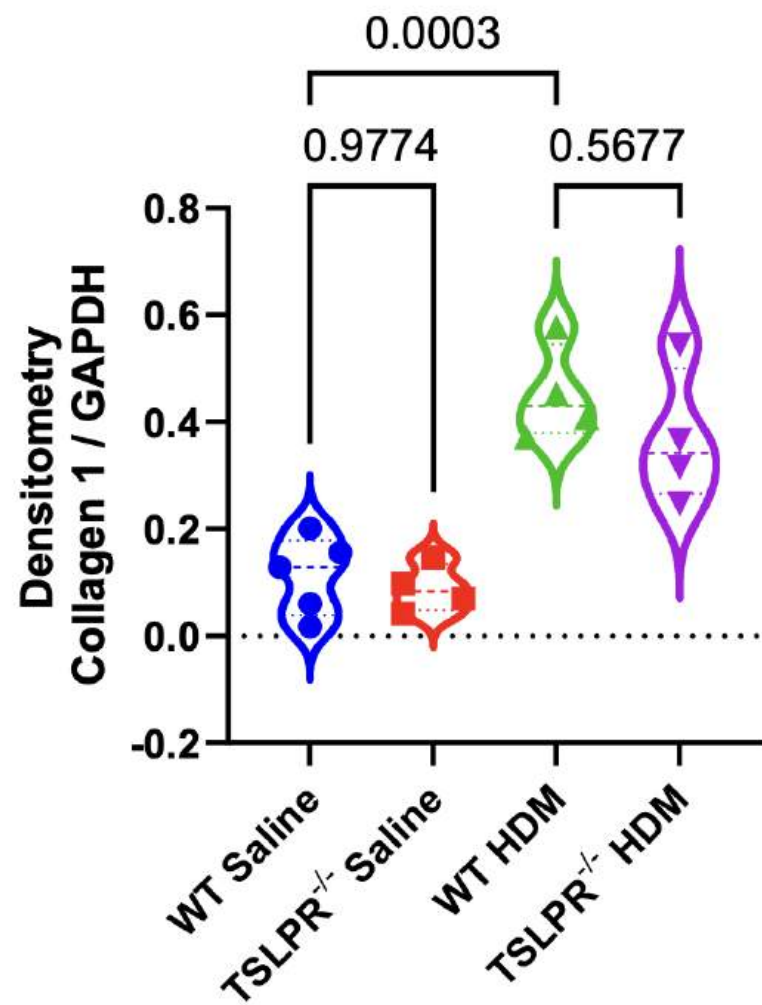**C**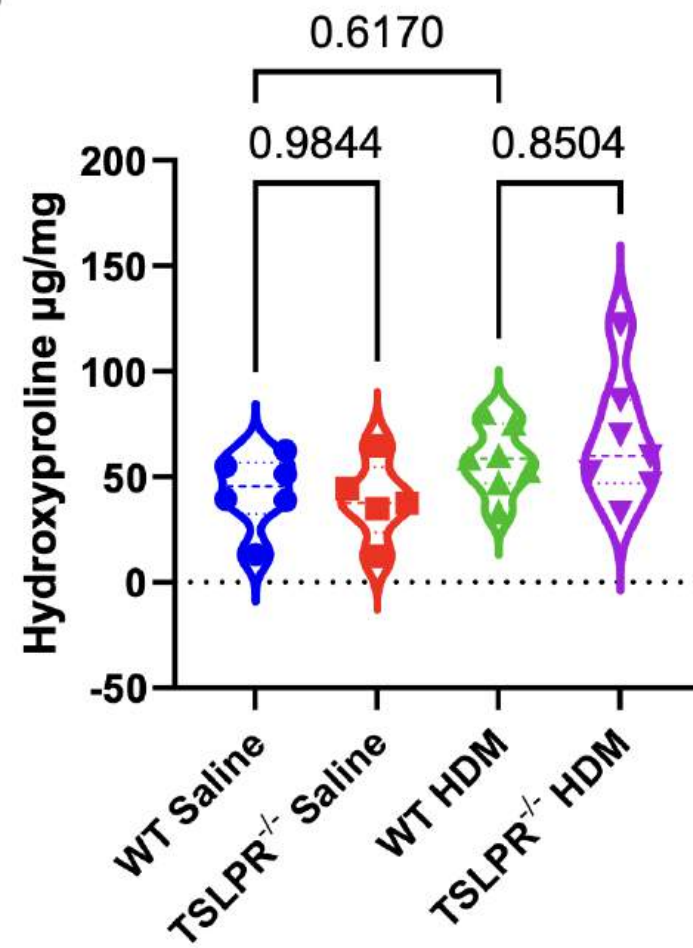

Supplement: S3 Fig — (A) The lung tissue lysates were subjected to immunoblot analysis with specific antibodies against collagen 1 and glyceraldehyde 3-phosphate dehydrogenase (GAPDH). (B) Expression level of collagen 1 was quantifies by densitometry and normalized with GAPDH. (C) Whole lung hydroxyproline levels in mice. Data are represented as the mean±SEM. Means with different superscript letters are significantly different from one another (*P < 0.05). Results are shown as mean ± SEM of three independent experiments. (PDF) [file pone.0335742.s003.pdf]
