# Supplementary material for: TSLPR deficiency attenuates AHR independently of eosinophilia and mucus secretion in a chronic HDM mouse model of allergic asthma
Source: PLoS One. 2025 Nov 19;20(11):e0335742. doi: 10.1371/journal.pone.0335742 (PMC12629416; doi:10.1371/journal.pone.0335742)
Supplement: Attachment — Submitted filename: Response to Reviewers .docx [file pone.0335742.s005.docx]

**Response to Reviewer 1:**

We thank the reviewer for taking the time to review our manuscript and for their constructive comments. We have addressed each of the suggestions in detail below and have revised the manuscript accordingly.

*Comment 1:*

“The paper is hard to follow because it lacks focus. This is especially true in the Discussion section. For example, the second paragraph of the Discussion is difficult to understand — it’s not clear what the main message is. The third paragraph seems to talk about AHR but also mentions cytokines and smooth muscle without a clear point. Each paragraph in the Discussion should focus on one idea and be rewritten more clearly.”

*Response 1:*

We have thoroughly revised the Discussion to enhance its clarity and organization. Specifically, each paragraph has been restructured to focus on a single, coherent idea. The second and third paragraphs have been rewritten to clearly separate the findings related to AHR, Th2 cytokines, smooth muscle cell responsiveness, and the feed-forward loop involving IgE and TSLP. Transitional sentences were added to improve logical flow between sections. Redundant content was removed to ensure focus on the main findings and their interpretation. We believe these changes significantly improve the readability and coherence of the Discussion.

*Comment 2:*

“The paper is too long. The Introduction, Results, and Discussion all need to be shortened. Please focus on the main points and cut out less important details.”

*Response 2:*

The Introduction was reduced by removing redundant background information and by summarizing complex pathways more succinctly. The Results section was streamlined by merging overlapping statements, condensing figure legends, and eliminating excessive methodological repetition. The Discussion was reduced as per your suggestion, while retaining all scientific findings and references. We ensured that each paragraph delivers a focused message with minimal redundancy.

*Comment 3:*

“Additional Experiments. More explanation about the lack of difference between TSLP knockout and wild-type mice would help. It would be interesting if the authors could explain more clearly why there is no difference in airway inflammation (such as type 2 cytokines or eosinophil infiltration) between the two types of mice. If possible, I suggest measuring IL-25 and IL-33 in this model.”

*Response 3:*

We thank the reviewer for this valuable comment. However, we respectfully clarify that, contrary to the reviewer's impression, our study does show a significant reduction in type 2 cytokines, including IL-4, IL-5, and IL-13, in both BALF and lung homogenates of TSLPR-deficient mice compared to wild-type controls (Figure 3A-G, S2). What remained unchanged were eosinophilic infiltration and structural remodeling features, such as mucus production and collagen deposition.

This apparent dissociation between cytokine levels and cellular inflammation suggests that compensatory epithelial cytokines, particularly IL-33 and IL-25, may sustain eosinophilia and remodeling even in the absence of TSLP signaling. Several studies support this possibility. For example, Chu DK et al. demonstrated that IL-33 is indispensable, while TSLP is dispensable, for Th2 responses during chronic allergen exposure (PMID: 23006545).

While we agree that measuring IL-25 and IL-33 would strengthen this hypothesis, such analysis would require a longitudinal, time-resolved study design, which falls outside the scope of the current work. We have now clarified these mechanistic considerations in the revised Discussion and suggested IL-33/IL-25 profiling as an important direction for future research.

**Response to Reviewer 2:**

***Comment 1:***

**Do murine ASM cells express Fc epsilon RI in the present model? (suggestion: flowcytometry of ASM cells collected from mice with or without the chronic HDM exposure, or immunohistochemistry)**

*Response 1:*

**We thank the reviewer for this important question. While FcεRI expression on human airway smooth muscle (ASM) cells has been demonstrated, including in our previous work showing functional responses to IgE stimulation (PMID: 16081836), the expression of FcεRI on murine ASM has not, to our knowledge, been clearly established. In mice, FcεRI is well characterized on mast cells, basophils, and certain dendritic cell subsets, but evidence for its expression on structural cells such as ASM is lacking.**

**Determining whether murine ASM expresses FcεRI would require cell-specific isolation and flow cytometric or immunohistochemical analysis with validated markers, which was beyond the technical and conceptual scope of this manuscript. Our current study focuses on the functional consequences of TSLPR deletion in vivo, including AHR, Th2 cytokines, and remodeling, rather than dissecting cell-specific receptor expression.**

**We agree that this is an interesting and important avenue for future investigation, and we have now acknowledged this limitation and future direction in the revised discussion.**

***Comment 2 & 3***

**Do murine ASM cells express TSLP in the present model? (suggestion: mRNA expression in ASM cells collected from mice with or without the chronic HDM exposure)**

**Do murine ASM cells express TSLPR in the present model? (suggestion: flowcytometry of ASM cells collected from mice with or without the chronic HDM exposure, or immunohistochemistry)**

*Response 2 & 3.*

**We appreciate the reviewer’s thoughtful questions. While our previous work demonstrated that human ASM cells express both TSLP and TSLPR (PMID: 17513456; PMID: 23892442; PMID: 21148792 and PMID: 20483734) similar data in murine ASM are lacking. Determining TSLP or TSLPR expression in mouse ASM would require specific cell isolation and characterization, which was beyond the scope of this study. We agree that this is an important direction for future research and have noted it in the revised discussion.**

***Comment 4:***

**Does the model depend on mast cells and basophils? (suggestion: deficient mice with the BALB/c background)**

*Response 4:*

**We thank the reviewer for this excellent suggestion. Dissecting the role of mast cells in the context of TSLPR deficiency would indeed require a complex model, such as a TSLPR/Mcpt5-Cre or Cma1-DTR double knockout (see PMID: 33725574). While we agree this would provide valuable mechanistic insight, it falls beyond the scope of the current study. We have now acknowledged this point in the revised discussion as a future direction.**

***Comment 5:***

**How does short period treatment of wild-type mice with anti-CD4 treatment (ex. last 1 week or shorter) affect airway inflammation and AHR? It may decrease Th cell-mediated airway inflammation but may not significantly affect HDM-specific IgE levels and TSLP levels in BALF and lung tissue, which may lead to IgE-mediated increase of AHR (via mast cell mediator stimulation or IgE direct stimulation of ASM cells).**

*Response 5:*

**We appreciate the reviewer’s mechanistic perspective. Several studies have addressed this question using anti-CD4 monoclonal antibody treatment. For example, Dakhama et al. (PMID: 12642393) showed that short-term anti-CD4 treatment significantly reduced airway hyperresponsiveness, BAL eosinophilia, Th2 cytokines (including IL-13), IgE levels, goblet cell hyperplasia, and subepithelial fibrosis. Similar findings were reported in other studies (PMIDs: 12626589, 18502692, 21778663), demonstrating that CD4+ T cell depletion suppresses both inflammation and remodeling features in allergen-challenged mice.**

**While this is an informative model, our current study focused on the genetic deletion of TSLPR and its effects across multiple readouts. Including an additional anti-CD4 treatment arm would involve a different experimental framework and was beyond the scope of this work. We have noted this as a potential complementary strategy in the revised discussion.**

***Comment 6:***

**What is the cause for the independency on TSLP-TSLPR pathway in the present 7-week model but not the conventional 5-week model? IL-33 or IL-25 might be upregulated after 5 weeks to 7 weeks. (suggestion: expression time course of TSLP, IL-33, and IL-25 during the HDM-inhalation period)**

*Response 6:*

**We thank the reviewer for this important point. We agree that compensatory upregulation of IL-33 or IL-25 during prolonged allergen exposure may explain the reduced dependency on TSLP-TSLPR signaling observed in our 7-week model compared to shorter protocols. This hypothesis is supported by studies showing increased IL-33 expression during extended HDM exposure, including work by Chu et al. (PMID: 23006545), who demonstrated that IL-33 is indispensable, while TSLP is dispensable, for driving Th2 responses during chronic allergen challenge.**

**This apparent dissociation between cytokine levels and cellular inflammation suggests that epithelial-derived cytokines such as IL-33 and IL-25 may help sustain eosinophilia and tissue remodeling in the absence of TSLP signaling. While we agree that a time-course analysis of these mediators would provide mechanistic insight, such longitudinal profiling was beyond the design and scope of the present study. We now address this point explicitly in the revised discussion (line 213) and have noted it as an important direction for future research.**

***Comment 7:***

**In the legends of Figs 2-5, S2, and S3, there is no description on the reproducibility of the results. It should be described in each of the legends.**

*Response 7:*

**Corrected. We have added a brief statement to each relevant figure legend indicating that the experiments were independently repeated with consistent results.**

***Comment 8:***

**According to the Methods, BALB/c background mice (with white color) were used for all the experiments. Why is the mouse in the Figure 1A black?**

*Response 8:*

It was corrected accordingly
