# Supplementary material for: TSLPR deficiency attenuates AHR independently of eosinophilia and mucus secretion in a chronic HDM mouse model of allergic asthma
Source: PLoS One. 2025 Nov 19;20(11):e0335742. doi: 10.1371/journal.pone.0335742 (PMC12629416; doi:10.1371/journal.pone.0335742)
Supplement: Attachment — Submitted filename: Response_to_Reviewers__auresp_2.docx [file pone.0335742.s006.docx]

**Response to Reviewer 2:**

We thank the reviewer for taking the time to review our manuscript and for their constructive comments. We have addressed each of the suggestions in detail below and have revised the manuscript accordingly.

*Comment 1:*

*“Additional experiment: According to their response letter, they will not perform new animal experiments to compare TSLP , IL33 and IL-25 production between the conventional 5-week model and their present 7-week model. The Reviewer can understand the reason that it is time consuming. However, the Reviewer expects that the authors can easily measure TSLP, IL-33, and IL-25 in BALF and lung by ELISA using the same samples used for Fig, 3A-D (BALF) and 3E-I (lung) collected in the previous experiments. It will not take for a long period to complete it. The data will be important to support their hypothesis to explain no decrease of airway inflammation in the TSLPR-deficient mice..”*

*Response 1:*

We thank the Reviewer for this important suggestion. We have now measured TSLP, IL-33, and IL-25 in both BALF and lung homogenates using the same samples as in Fig. 3A–D and Fig. 3E–I. These results are included in the revised manuscript (new Figure 3J–N). We found reduced IL-33 and increased IL-25 levels in TSLPR^-/-^ mice compared to WT, while TSLP showed no significant difference. These findings support our conclusion that IL-25 and IL-33 may act as compensatory alarmins in the absence of TSLPR signaling.

*Comment 2:*

*“Line 200: The authors need to clearly describe that expression of TSLPR, TSLP, and Fc epsilonRI had been reported in human ASM cells but still unknown in murine ASM cells. This is important point not to mislead the readers. The reviewer feels that the end of this paragraph would be the best place where they describe it.”*

*Response 2:*

Revised as suggested. We now state that expression of TSLPR, TSLP, and FcεRI has been reported in human ASM cells, but remains unknown in murine ASM cells.

*Comment 3:*

*“Lines 216/217: The sentence will be read as that “their (IL-33 and IL-25) upregulation in the TSLPR-deficient mice” had been reported already. Is it true?”*

*Response 3:*

The sentence has been rephrased to clarify.

*Comment 4:*

*“Lines 73-75: The authors need to describe that they demonstrated it for human ASM cells (but unknown for mice). “ASM” to be “human ASM” in the line 74. Line 192: “ASM” to be “human ASM cells”. Line 193/194: “likely reflects” to be “might reflect”. Line 197: “ASM cells”to be “human ASM cells”. These specifications are necessary not to mislead the readers.”*

*Response 4:*

All relevant mentions have been changed to “human ASM cells,” and “likely reflects” has been revised to “might reflect.”

*Comment 5:*

*”Line 85: “eight weeks” is wrong. To be changed to “seven weeks”. Line 92: “8 weeks” is wrong. To be changed to “7 weeks”*

*Response 5:*

Corrected.

*Comment 6:*

*“ Fig. 1A: According to the illustration, the last i.n. administration was done on the day 50. Is it correct? In lines 257-260 of the Methods section, the authors described that the administration was for the 7 weeks, and it could be read as that the last administration was done on the day 47. What was done on the day 50? The same amount of i.n. administration with the day 47?”*

*Response 6:*

Clarified. The final HDM administration was performed on day 47, and mice were sacrificed on day 50.

*Comment 7:*

*“Fig. 1B-D: Specify the day number for the data collection. According to the lines 257-260 of the Methods section, it would be the day 52 if the day 50 was the last administration or the day 49 if the day 47 was the last administration.”*

*Response 7:*

Legends revised to specify that lung function data were collected on day 50.

*Comment 8:*

“*Line 114: Is the description correct for the violin plots of the Fig. 2 C, E-H and the scattered plot with not error bars of the Fig. 2D?”*

*Response 8:*

Corrected. The legend now accurately describes the violin plots (Fig. 2C, E–H) and scatter plot (Fig. 2D).

*Comment 9:*

“*Lines 96/97, 114, 132, 146, 168: Which do the last sentences of the Figure legends mean that the authors showed all the data of the 3 independent experiments or data from only 1 selected experiment among the 3 independent experiments with similar results?”*

*Response 9:*

All the data of the 3 independent experiments were included in the figures.

*Comment 10:* “*Line 349: There is no indication with *, ** and *** despite the statistical differences in all the Figures”.*

*Response 10:*

Revised.
