# Supplementary material for: TSLPR deficiency attenuates AHR independently of eosinophilia and mucus secretion in a chronic HDM mouse model of allergic asthma
Source: PLoS One. 2025 Nov 19;20(11):e0335742. doi: 10.1371/journal.pone.0335742 (PMC12629416; doi:10.1371/journal.pone.0335742)
Supplement: Attachment — Submitted filename: Response_to_Reviewers__auresp_3.docx [file pone.0335742.s007.docx]

**Response to Reviewer 2:**

We thank the reviewer for taking the time to review our manuscript and for their constructive comments. We have addressed each of the suggestions in detail below and have revised the manuscript accordingly.

Comment 1:

Line 358 and all the legends of Figures and Supplementary Figures: Is the description “The data were indicated as mean ± SEM” correct for the violin plots? Isn’t it to be “median and quartiles” in violin plots by the GraphPad Prism software?

Response:

We thank the reviewer for catching this important detail. You are correct that in GraphPad Prism, violin plots display the median and interquartile range rather than mean ± SEM. We have corrected the figure legends accordingly to accurately reflect the data representation. The revised text now reads:

Comment 2:

Figure 2D: The bars for the means and the SEMs are hard to see.

Response:

We appreciate this observation. We have enhanced the visibility of the mean ± SEM bars in Figure 2D by adjusting line thickness and color contrast in GraphPad Prism to ensure they are clearly visible in both print and digital formats.

Comment 3:

Figure 2: The location of the panels to be improved for readability. The BALF TSLP and IL-33 panels (J and K) to just follow panels for BALF Th cytokines (A-D). Lung TSLP, IL-33, and IL-35 panels (L-N) to just follow panels for lung Th cytokines (E-H).

Response:

Thank you for this helpful suggestion. We have rearranged the panels in Figure 2 to follow the reviewer’s proposed order, improving logical flow and readability.

Reviewer Comment 4:

Line 220: “TSLP-IL-33 signaling” to be “TSLP signaling and attenuation of IL-33 signaling.”

Response:

The phrase has been revised to “TSLP signaling and attenuation of IL-33 signaling” in line 220 to better reflect the mechanistic distinction between these pathways.
